# Civil war as a social process: actors and dynamics from pre- to post-war

**DOI:** 10.1177/13540661221095970

**Published:** 2022-06-06

**Authors:** Anastasia Shesterinina

**Affiliations:** The University of Sheffield, UK

**Keywords:** Civil war, actors, dynamics, interaction, social process, conceptual framework

## Abstract

What accounts for overarching trajectories of civil wars? This article develops an account of civil war as a social process that connects dynamics of conflict from pre- to post-war periods through evolving interactions between nonstate, state, civilian, and external actors involved. It traces these dynamics to the mobilization and organization of nascent nonstate armed groups before the war, which can induce state repression and in some settings escalation of tensions through radicalization of actors, militarization of tactics, and polarization of societies, propelled by internal divisions and external support. Whether armed groups form from a small, clandestine core of dedicated recruits, broader networks, social movements, and/or fragmentation within the regime has consequences for their internal and external relations during the war. However, not only path-dependent but also endogenous dynamics shape overarching trajectories of civil wars. During the war, armed groups develop cohesion and fragment in the context of evolving internal politics, including socialization of fighters, institution-building in the areas that they control, which civilians can collectively resist, competition and cooperation with other nonstate and state forces, and external influence. After the war, armed groups transform to participate in continuing conflict and violence in different ways in interaction with multiple actors. This analysis highlights the contingency of civil wars and suggests that future research should focus on how relevant actors form and transform as they relate to one another to understand linkages between conflict dynamics over time and on continuities and discontinuities in these dynamics to grasp overarching trajectories of civil wars.

What accounts for overarching trajectories of civil wars? The dominant form of armed conflict since World War II, civil wars have attracted significant academic attention.^1^ Early quantitative studies examined the distinct phases of onset, duration, and termination of civil war, with a focus on civil war recurrence and other challenges of post-war recovery in the aftermath. Looking at factors that affect outcomes at the macro-level, this research formed the basis for our understanding of the structural determinants of civil war. More recent turns in the literature to the micro-foundations of individual and group behavior, organizational structure, and legacies of civil war have seen increasingly sophisticated research designs with the use of fine-grained data collected and analyzed at the subnational level. Scholars have asked questions of not only *why* and *to what extent* but also and primarily *how* and enriched our understanding of the dynamics of civil war. By extending the scope of analysis beyond binary start and end points, expanding the range of actors beyond state-nonstate dyads, and shifting the focus from factors to dynamics, this literature has started to depict civil war as a process with blurry boundaries between pre-war, wartime, and post-war periods of conflict. Critical interventions on peace and conflict have reaffirmed this trend. While a consensus has emerged on the continuing need to bridge the different phases and explanatory logics of civil war (Cederman and Vogt, 2017), questions remain about how to explore such linkages. How do civil wars unfold from pre- to post-war? How do different dynamics of civil war shape one another and change over time? How should we approach civil war in processual terms?

This article contributes to the efforts to grapple with the complexity of civil war by developing an account of civil war as a social process that incorporates dynamics of conflict from pre- to post-war periods. These dynamics, I argue, unfold through evolving interactions between the actors involved. Multiple nonstate, state, civilian, and external actors, which are more or less relevant for specific dynamics, form and transform as they relate to one another in the context of conflict. The dynamics that their interactions engender emerge at different points in the conflict, intersect, and shift over time to shape overarching trajectories of civil wars in path-dependent and endogenous ways. Tracing these dynamics from pre- to post-war periods can help illuminate central questions in the study of civil war, namely, how armed conflict originates in different ways and how these origins condition the progression of civil wars and impact war-to-peace transitions together with wartime and post-war developments.

In adopting this actor-centered and relational, in other words, *social* approach to the process of civil war, the article draws on the literature on contentious politics and advances calls to put civil war studies in closer conversation with this literature (Tarrow, 2007; Wood, 2015). This literature recognizes the contingent character of actors and their interactions, problematizing the linear progression of contention, but seeks to identify regularities in trajectories of contention and views recurrent mechanisms, such as brokerage, as a source of these regularities (McAdam et al., 2001). I similarly start from a nonlinear view of civil war, where it is not predefined actors engaging in interactions that set off predictable sequences of events but multidirectional and changing relations that can turn the process in unexpected ways. In contrast to the contentious politics tradition, I look at broader dynamics that have been identified as common during pre-war, wartime, and post-war periods in scholarship on the dynamics of civil war and that can contain multiple mechanisms. For example, the dynamics of mobilization involve brokerage alongside other mechanisms. Maintaining an analytical distinction between pre-war, wartime, and post-war periods while appreciating that these periods need not take place one after the other when tracing these dynamics can help understand the linkages in these dynamics over time. While the dynamics that I outline are not exhaustive, this first effort in the literature to chart key dynamics across periods of conflict is a fruitful starting point in locating such linkages in particular and in capturing how different dynamics combine into the social process of civil war in general.^2^

These dynamics are evident before the war in the mobilization and organization of nascent nonstate armed groups. Whether they emerge from a small core of dedicated individuals using clandestine methods, broader networks, social movements, and/or splits within the regime, these groups require civilian support, from recruits to secrecy to provision of resources and hiding places, in order to survive. Social ties and identities that link these groups to their internal and external bases of support can form in the pre-war period, including in the course of observation of and participation in everyday confrontation, nonviolent contention, and violent opposition. These repertoires of collective action are often met with state repression and can escalate to war through radicalization of actors, militarization of tactics, and polarization of societies. However, not all civil wars escalate from intensifying or widening collective action and state-society interaction—some break out unexpectedly, whereas others follow prolonged periods of low-intensity violence. Moreover, social ties and identities can transform and new ones can develop during the war.

One of the linkages between pre-war and wartime periods, therefore, is the influence that mobilization and organization of nonstate armed groups and their bases of support have on these groups’ internal structure and their relations with nonstate, state, civilian, and external actors. For example, social embeddedness of nonstate armed groups in local populations can have important consequences for the cohesion of these groups and their predatory behaviors toward populations. While pre-war configurations can have such path-dependent effects, civil war trajectories can evolve in endogenous ways as a result of the intersecting dynamics of intragroup socialization through training, political education, and participation in violence, violence against civilians, and institution-building in the areas where nonstate armed groups establish territorial control, which civilians respond to in different ways, competition and alliance formation among nonstate armed groups, their conflictual and cooperative relationships with various state actors, and external influence.

These dynamics leave long-lasting legacies in the post-war period, but not all post-war outcomes are directly related to civil war and some can be driven by pre-war dynamics, combined wartime and post-war effects, and altogether new dynamics that emerge after the war. Here the focus is on armed actors’ disarmament, demobilization, and reintegration, including through power-sharing at the top level. Still, belligerents often return to organized violence and new armed actors and violent activities appear. Social ties and identities continue to transform and groups affected by wartime and post-war violence, among others, mobilize alongside international efforts to maintain their activities amid changing local circumstances where peacebuilding institutions are co-opted by spoilers in and outside of the regime. As old and new forms of conflict develop in these environments, with former, remaining, and emergent armed groups reconfiguring their relations with other nonstate and state actors and continuing to exercise control over civilian populations, the linkages between dynamics across pre-war, wartime, and post-war periods of conflict come to the fore.

Because wartime dynamics are intricately related to pre-war and post-war periods, as this article demonstrates, scholarship on civil war should move further toward a processual view of civil war. Recent work has operationalized this move in a number of ways. For example, quantitative studies have introduced two-stage models to understand whether factors associated with civil war onset, such as horizontal inequalities, have different effects on the emergence of nonviolent and violent conflict short of war in the first stage and on the escalation of the conflict to armed violence in the second (e.g. Bartusevičius and Gleditsch, 2019; Cunningham et al., 2017; Germann and Sambanis, 2021). These studies have shown that nonviolent and violent conflict indeed often escalates to civil war and that variation in civil war onset in countries with factors associated with this outcome is due to the different effects these factors have on the two stages of conflict escalation. This two-stage approach, however, misses those cases that do not follow a linear pattern of escalation and does not give us a sense of how pre-war mobilization and organization of nascent nonstate armed groups and state-society interaction shape the evolution of civil wars once armed violence sets on.

An understanding of overarching trajectories of civil wars requires advancing the analysis beyond a focus on individual dynamics in a single period, such as pre-war escalation, or how individual dynamics, such as mobilization, change throughout conflict and on the linkages between some but not other dynamics across some but not other periods, such as between wartime violence and post-war political participation. The approach to civil war as a social process connecting these dynamics through evolving interactions between internal and external actors involved is a step in this direction. It invites scholars of civil war to focus on how actors form and transform as they relate to one another in order to trace dynamics from pre- to post-war periods of conflict and on continuities and discontinuities in the dynamics that their interactions generate in order to grasp overarching trajectories of civil wars. Developing grounded knowledge of these actors’ own perceptions of their reality through close, ethical engagement with primary and secondary materials in single and comparative case studies of civil war is the methodological foundation of this approach.

In the following sections, I, first, expand on the notion of civil war as a social process. I then detail key pre-war, wartime, and post-war dynamics that nonstate, state, civilian, and external actors jointly produce through their interactions and that scholars should pay attention to when exploring overarching trajectories of civil wars in specific cases. I refer to illustrative examples throughout the discussion. The article concludes with implications of this approach for research and policy and ways forward for future studies of civil war.

## Civil war as a social process

Studies in sociology, political science, and anthropology have advanced the notion of civil war as a social process. Writing about a wide range of contentious politics, from revolutions to civil wars to social movements, McAdam et al. (2001: 24) argue that contentious politics involves complex social processes and view such processes as regular sequences and combinations of causal mechanisms, which they define as “a delimited class of events that alter relations among specified sets of elements in identical or closely similar ways over a variety of situations.” Brokerage is one such mechanism, which entails “the linking of two or more previously unconnected social sites by a unit that mediates their relations with one another and/or with yet other sites” (p. 26). Focusing on how actors formed and transformed as they interacted in the lead-up to the American Civil War, for example, the authors show that brokerage of a coalition between anti-slavery Northerners and free-soil-seeking Westerners combined with other mechanisms to produce the onset of this war. The authors also show that such combinations are contingent and might not lead to the same outcome under different circumstances, as in the case of the Spanish transition to democracy.

While also looking at recurrent causal mechanisms, such as alliance, which unlike brokerage does not presume common preferences and entails “an exchange between local and supralocal actors” whose preferences differ in civil wars, Kalyvas (2006) argues against studying civil war within the broader field of contention because “war and peace are radically different contexts that induce and constrain violence in very different ways” (pp. 14, 22). Instead, he makes an analytical distinction between violence and war, focusing on violence committed intentionally against non-combatants in irregular wars where conventional state militaries face lightly armed rebels, and conceptualizes such violence as “a dynamic process” (p. 22). Here sequences of decisions, actions, and events intersect to produce violence involving (often invisible) civilians who are neither victims nor perpetrators but who partake in this process by providing information to political actors. This insight is evident in the Greek Civil War and other cases of irregular war. From this perspective, civil war is “a [broader] process that connects the collective actors’ quest for power and the local actors’ quest for local advantage” (p. 383). It is a “social process” because of the centrality of “strategic interaction between rival actors, and between these actors and the population,” in the dynamics of territorial control that shape the evolution of civil wars (Kalyvas, 2008a: 1061, 1063). Civil war is relatedly an endogenous process where “behavior, beliefs, preferences, and even identities can be altered as a result of the conflict and its violence” (Kalyvas, 2008b: 403).

Wood (2003) highlights this endogeneity of preferences to the process of participation in high-risk collective action during the war. By supporting the insurgency, *campesinos* in El Salvador experienced what Wood calls “pleasure of agency,” which reinforced an emergent insurgent political culture and set further collective action in motion, which once again reinforced insurgent values, norms, beliefs, practices, and support networks, forging new opportunities for collective action (p. 18). A collective identity as members of the insurgent community—a part of the new political culture—was, thus, the outcome of collective action and the process connecting the two was both endogenous and recursive as “the cycle of action, success, pleasure in agency, and reinforcement of insurgent culture recurred” (p. 238). Such “transformation[s] of social actors, structures, norms, and practices” are what Wood subsequently called “the social processes of civil war” (Wood, 2008: 540). These processes, from political mobilization to transformation of gender roles, which I refer to as “dynamics” to differentiate this category from the encompassing notion of civil war as a “social process,” can leave enduring legacies, according to Wood, and this connects civil war to the post-war period. Such dynamics, however, do not all originate during the war and some can be traced to the pre-war period, even though wartime and post-war developments can change the course and consequences of these dynamics.

As Lubkemann (2008: 324, emphasis in original) argues, “rather than treating war as an interruption of social process,” we need to recognize that social relations develop dynamically “*throughout* conflict.” Theorizing the social condition in war through a focus on wartime mobility in Mozambique, Lubkemann demonstrates that dynamics other than violence, including pre-war dynamics, shape wartime social process, including violence itself, and involve a broader range of actors than political actors struggling for power. He shows, for example, that “local social agendas—rooted in prewar social tensions and culturally speciﬁed logics—strongly inﬂuenced the deployment of violence by FRELIMO and RENAMO from the very outset of the conﬂict” (p. 175). Likewise, Staniland (2014: 9) finds that “prewar politicized social networks” shape the nature of rebel organizations that develop in civil wars and constrain their wartime activities. Such networks can be seen as part of what Weinstein (2007: 7) refers to as “social endowments”—the “shared beliefs, expectations, and norms” that exist in communities and that nonstate armed groups can exploit—which along with “economic endowments,” from natural resource extraction to external patronage, affect their violent strategies during the war. The importance of pre-war social relations, networks, and other resources for wartime that these authors emphasize, working in different anthropological and political science traditions, indicates that not only endogenous but also path-dependent dynamics underlie the process of civil war.

Scholars of contentious politics have made a similar argument in relation to civil war. In general, civil war has been studied in isolation from the field of contentious politics, yet nonviolent and violent forms of contention, from demonstrations to clashes, typically precede and can be meaningfully related to civil war. A range of civil wars in cases as diverse as the former Yugoslavia and Syria grew out of intense contention over democratization, for example (Della Porta et al., 2018). Such pre-war contention plays important roles in the formation of “collective conflict identities” that Shesterinina (2021: 2) finds in the case of Abkhazia emerged before rather than during the war and were central to the mobilization and organization for war. This means that in order to understand civil war “in processual terms we must first comprehend the practices of war and peace: how people mobilize and organize for war, and the role played by ideational factors in such mobilization and organization” (Richards, 2005: 13). But we also need to understand how pre-war dynamics of mobilization and organization affect wartime interactions between rival armed forces, local populations, and other actors involved and how wartime dynamics, in turn, shape post-war opportunities for peace as conflict and violence continue in old and new ways.

As Campbell et al. (2017: 92–93) show, “[d]uring civil war, conflict and peaceful cooperation coexist and coevolve” and, “during periods of supposed peace, violent conflict often continues,” resulting in contexts that are “neither full war nor durable peace.” These contexts, from Burundi to Colombia, have been variously termed “no peace, no war” (Richards, 2005), “peace in between” (Suhrke and Berdal, 2012), and “peaceandconflict,” where peace and conflict are “intertwined and co-constitutive” (Mac Ginty, 2022: 51), to capture the heterogeneous nature of civil wars. Conflict-mitigating and conflict-disrupting opportunities, such as armed actors’ turn to nonviolent strategies (Dudouet, 2015), exist across these contexts, which reiterates the contingency of the underlying process of civil war. As Mac Ginty (2022) explains, conflict disruption may or may not produce intended change as it is embedded in a complex system where multiple actors interact simultaneously.

Civil war as a social process, therefore, connects the pre- to post-war dynamics of conflict through changing relations between the actors involved within the social settings in which they interact but does not presuppose a particular combination or sequence of dynamics as some actors can become more or less relevant in shaping these dynamics at different points in time and different dynamics can, as a result, emerge, shape one another, and shift in unexpected ways. These actors, Mampilly (2011) finds are state and nonstate armed forces, including rival nonstate armed groups, civilian populations, from ordinary inhabitants of the areas that armed actors control to traditional and religious leaders to civil society groups, and external actors, such as international organizations, humanitarian agencies, and neighboring states, among others. Their interactions start before the war, with efforts by nascent nonstate armed groups to mobilize support, recruit participants, and organize their activities. The state seeks to repress these efforts and this response can escalate tensions, radicalizing actors, militarizing tactics, and polarizing societies, sometimes as a result of splits within relevant actors and external support. During the war, these pre-war relations underpin civilian support for different actors and armed groups devote considerable resources to the socialization of fighters and establishing political control, including through institution-building, which some civilian populations collectively resist. Some armed groups develop cohesion, whereas others fragment as a result of internal divisions, competition with rivals, counterinsurgency, and/or external intervention. After the war, these dynamics leave legacies for the transformation of armed actors—and conflict and violence,—political participation of social groups affected by war, and continued external influence.

As a result, in contrast to conventional definitions of civil war that quantitatively establish whether a case qualifies as a civil war or not based on annual battle-death thresholds, among other criteria (Sambanis, 2004), or those that qualitatively distinguish civil wars from other forms of contention, especially pre-war contention (Kalyvas, 2006), the processual approach to civil war underscores that civil war does not set on or end with the outbreak or termination, respectively, of combat violence of particular intensity or character between nonstate and state armed forces in pursuit of political control over the state or part of its territory. Instead, it extends into the period before such violence develops and after it stops or changes in character and incorporates a range of pre- to post-war nonviolent and violent dynamics that combine into the process of civil war.

## Mobilization and organization for war

The dynamics of mobilization and organization lie at the heart of this process. While nonstate armed groups require only a small number of committed individuals to launch insurgency under the right conditions (Fearon and Laitin, 2003), no armed group can survive without mobilizing support, recruiting participants, and organizing its activities toward even loosely defined goals (Wickham-Crowley, 1992). Building on the collective action tradition, scholars of civil war mobilization start from the recognition that participation in insurgency is risky and potential participants will want to benefit from the insurgency’s success without contributing to this outcome—a classic “free-rider” problem (Viterna, 2013: 42). They stress selective incentives that existing armed groups offer to recruit participants (Humphreys and Weinstein, 2008; Weinstein, 2007), social norms that induce individuals to accept high risks, especially in strong communities (Petersen, 2001), and emotional rewards of participation (Wood, 2003), among other, for example, ideological motivations (Sanín and Wood, 2014).^3^ In turn, studies of organization stress the importance of control that armed groups impose on fighters to constrain and direct their activities (Staniland, 2014; Weinstein, 2007), including in situations of forced recruitment (Gates, 2002), and specific institutions, such as political education, these groups develop to establish internal control (Hoover Green, 2018).

Yet before the war differences exist in how nascent armed groups mobilize and organize. One dynamic Lewis (2020) charts is that of rebel groups that form without internal material resources or external support and initially mobilize a small following of carefully vet recruits to organize clandestine activities against the state. In weak states where governments seek information on rebellion from civilians, civilian support entails secrecy and nascent groups induce civilians to keep their activities secret from state actors by spreading rumors, which generates grievances against the state. They also engage in small-scale violence against easy state targets, which signals their potential to challenge the state. The few groups that become viable, in other words, that pose at least a minimal threat to the state, are those that manage to convince the population of their capacity to bring about change and this is particularly the case in ethnically homogeneous areas where dense networks of trust allow for the transmission of rebel messages. Uganda is an example where a number of rebel groups attempted forming, but only some, notably the Lord’s Resistance Army (LRA), became viable through this dynamic.

In another dynamic advanced by Staniland (2014), rebel groups emerge not from a small, clandestine core but from pre-war politicized networks that are not built for war. While unpoliticized groups, such as bowling leagues, and pro-state networks, such as paramilitaries, are unlikely to be mobilized for rebellion, politicized networks, particularly nonviolent opposition, including political parties, religious associations, and student activist groups, can be repurposed for rebellion. These social bases link organizers to one another through horizontal ties and organizers to local communities through vertical ties, enabling communication, coordination, and cooperation across and within communities that help overcome the challenges of transition from pre-war activities to training and funding fighters, creating coercive institutions, and engaging in violence. The Taliban in Afghanistan is an example of an integrated organization that combined robust central and local control in this way. But different types of organizations result from the variation in the social bases and most change over time. Importantly, Staniland finds actors of the kind Lewis describes that prepare for violence before the war, even when they aim to establish networks appropriate for war, rarely become dominant insurgent forces once war breaks out. This is not only because they ultimately attract state attention and are likely to be undermined before becoming viable, as Sullivan (2016) confirms, but also because their social bases can develop in unintended ways, for example, by spilling into mass protests or coups rather than insurgency.

Armed groups, however, also emerge from mass protests and coups. A dynamic Cederman et al. (2013) outline starts not with small, clandestine groups, as in Lewis’ account, or politicized networks, as in Staniland’s account, but with the politicization of group-level inequalities by intellectuals, dissidents, and political entrepreneurs, which generates emotionally charged grievances that propel mass mobilization.^4^ When states defend unequal arrangements and resort to indiscriminate repression in response, state challengers “have little choice but to arm themselves” and violence escalates (p. 50).^5^ Divisions within social movements (Seymour et al., 2016), defections from state repressive apparatuses that channel arms and military skills to nascent armed groups (Della Porta et al., 2018), and external support, whether actual in the form of arms, funds, recruits, and safe havens (Salehyan, 2009) or anticipated (Jackson et al., 2020), fuel radicalization of actors, militarization of tactics, and social polarization underpinning escalation (Florea, 2017). Civil wars in the former Yugoslavia are a notorious example of this dynamic.

Social movement organization is particularly important in this dynamic. As Pearlman (2011) argues, “when a movement is fragmented, it lacks the leadership, institutions, and collective purpose to coordinate and constrain its members” (p. 2). Indeed, the presence of groups with radical claims within movements (Vogt et al., 2021) and violence-wielding groups, such as radical flanks and parallel armies (Ryckman, 2020), increases the chance of escalation. From the bargaining perspective, this is because fragmentation affects not only what happens inside movements but also their perception by states. Internal divisions “exacerbate information problems and increase uncertainty for states about what concessions might satisfy the movements,” preventing credible commitments to potential settlements (Cunningham, 2013: 660).^6^ In contrast, cohesive movements “mobilize mass participation, enforce strategic discipline, and contain disruptive dissent” (Pearlman, 2011: 2). These are some of the main features of movements that succeed in bringing about the changes that they set out to achieve without recourse to violence. Mass popular support increases the cost of state repression, encourages nonviolent loyalty shifts among regime supporters, and offers movements diverse tactics, facilitating sustained pressure on the state (Chenoweth and Stephan, 2011). This differentiates cases where movements, such as the Palestinian national movement, turn to violence, as in the 1960s, from those where they do not, as early in the first Intifada.

Krause (2018) illuminates another side of escalation. In “non-state armed conflict[s] between social groups,” such as those in Ambon in Indonesia or Jos in Nigeria, communities that are vulnerable to armed conflict because of internal mobilization potential and the threat of attacks from external armed groups can become resilient through prevention efforts by local leaders and residents who establish social orders that support non-escalation (p. 6). These actors do so by “counter[ing] polarization and develop[ing] inclusive cross-cleavage identities; establish[ing] internal social control, persuading residents to support prevention efforts and formulating rules and procedures for conflict management; and engag[ing] external armed groups for negotiations and the gathering and dissemination of information” (p. 7). This allows communities to adapt to rapidly changing conflict environments at the outbreak of armed conflict and in its course. On the contrary, in communities that do not develop such preventive capacity, violence escalates from neighborhood-based pogroms to battles between armed groups and joint attacks of mobile gangs and militias with participation of civilians.

But not all pre-war contention *escalates* to civil war. Pre-war mobilization and organization can relate to civil war in ways that do not follow linear escalation. As Shesterinina (2021) shows, decades of participation in and observation of everyday confrontation, political contention, and violent opposition preceded the war in Abkhazia. Nonetheless, the war broke out abruptly, following years of relative calm when few violent events took place after the intergroup clashes that split the society and prompted the formation of armed groups on the Georgian and Abkhaz sides of the conflict. Violence, therefore, did not increase in intensity and scale, although nonviolent conflict continued before the war. Instead, the war began with the advance of Georgian forces into Abkhazia and the mobilization of the Abkhaz who were recruited into the Abkhaz Guard before the war and those who were not. Pre-war collective action, however, shaped shared understandings of the conflict and one’s role in it, or collective conflict identities that helped ordinary people make sense of and decide how to respond to the Georgian advance, or collectively frame it as a threat. It also laid the foundation for the organized and spontaneous mobilization that small groups bound by pre-war ties engaged in when the war began and the organizational capacity of the Abkhaz army that formed during the war based on preexisting structures and experience.

The focus on escalating state-society interactions also misses state challengers that emerge from within the regime as a result of coup d’état attempts (Roessler, 2016), army splinters not accompanied by coups (McLauchlin, 2022), and exclusion from the military (Harkness, 2018). In weak, ethnically divided states, Roessler (2016) illustrates, elites in the central government mobilize support from beyond their own ethnic base to extend the state’s reach. This, however, creates opportunities for rival elites to seize power in the future using their partial control of the state, above all the military. To reduce their rivals’ coup-making capabilities, leaders exclude rivals from the government when a threat appears, for example, through purges, but this increases the risk of civil war as the rivals mobilize armed groups from within their social bases to overthrow the government. This is particularly likely when leaders cultivate patronage networks (Reno, 2007) and use coercive institutions outside of military command, such as republican guards and secret police, to counterbalance the military (De Bruin, 2020). Mobilization and organization of armed groups resulting from fragmentation within the regime are evident in Sudan, Liberia, and Sierra Leone, among others.

## Armed groups’ internal and external relations

The different ways in which nonstate armed groups mobilize and organize for war impact their internal structure and interactions with other actors during the war. For example, preexisting networks that rebel groups are built on shape their cohesion and fragmentation. As Staniland (2014) argues, overlapping social bases that tie organizers to each other and local communities allow these organizations to establish a unified central authority and strong local institutions of command and control able to channel resources toward the goals of rebellion rather than opportunistic behaviors. Indeed, violence against civilians is common among armed groups that are not embedded in local communities (Weinstein, 2007). The LRA’s limited violence in the area where it formed and was “embedded in almost every village” in its early years and the horrific atrocities, including forced recruitment of child soldiers, it perpetrated later when civilian support waned are exemplary (Lewis, 2020: 119).

This case as well demonstrates that not only pre-war configurations but also wartime dynamics impact internal and external relations of nonstate armed groups. As Hoover Green (2018) finds, to avoid behaviors that threaten the armed group’s survival, leaders of armed groups with diverse mobilization and organization origins, from El Salvador to Sierra Leone, devote significant resources to political education, including ideological indoctrination, with the aim of changing their fighters’ intrinsic incentives for violence and thereby restraining unwanted violence. Other forms of fighter socialization exist, from formal training, initiation rituals, and hazing (Wood, 2008), which create systems of behavioral rules, punishments, and rewards intended to discipline fighters, to participation in violence in groups (Fujii, 2009), especially sexual violence, which bonds groups of previously unacquainted fighters in contexts of forced recruitment (Cohen, 2016).^7^ These dynamics characterize not only nonstate armed groups but also state forces (McLauchlin, 2020; Manekin, 2020).

The nature of rebel organizations that develop through these internal dynamics and interactions with other actors affects changing patterns of violence these groups engage in and their ability to withstand counterinsurgency and intergroup competition, in other words, “organizational resilience” (Parkinson, 2013: 418; Parkinson and Zaks, 2018). It also affects armed groups’ ability to build governance institutions in the areas they control. As Arjona (2016) shows, where rebels face internal indiscipline, external competition over territory, and broader changes in the war, for example, peace negotiations, they focus on short-term goals rather than on establishing social contracts in civilian communities and disorder characterizes their presence. In contrast, disciplined groups focused on long-term goals are able to establish complex systems of governance, taxing civilians, providing health, education, and police services, and instituting courts to adjudicate disputes. They create expectations among civilian populations and induce collaboration through institution-building.^8^ The ability of some but not other armed actors to rule different areas of Colombia exemplify this dynamic.

Civilians, however, are not simply recipients of rebel rule (Mampilly, 2011). Some flee the areas that armed groups control or collaborate with these groups in hopes of their protection, which is not guaranteed, whereas others collectively respond to and even resist the imposition of control by armed groups, whether coercive or institutional.^9^ Preexisting local institutions of dispute resolution (Arjona, 2016) and cooperation (Kaplan, 2017), which help communities overcome the collective action problem despite the fear of armed groups, are critical for resistance. Such institutions differentiate communities that retained their decision-making capacity in the areas that the Revolutionary Armed Forces of Colombia (FARC) controlled from those that did not. In particular, Kaplan (2017) finds that collective strategies of managing the internal order of communities, avoiding participation in the conflict, limiting armed groups’ access, and demanding accountability enable community resistance. These strategies include checking everyday conflicts among neighbors that armed groups could exploit, organized protest, and actions by unarmed community guards. Armed self-defense groups have also been identified among civilian responses (Jentzsch et al., 2015). These responses variously impact armed groups “by making killing more difficult or even costly. . ., by incorporating new ideas, or by affecting internal group politics” (Kaplan, 2017: 11).

Civilian responses, however, cannot be divorced from the broader environment where armed groups interact with the state, competing armed groups, and international actors, which can shift armed groups’ incentives over time and the ways in which these groups engage with civilians. While these interactions are often violent, differently so based on “technologies of rebellion” (Balcells and Kalyvas, 2014: 1391), armed groups find themselves in cooperative relations with the state, even in the absence of ceasefires and peace agreements (Campbell et al., 2017). Staniland (2012) identifies practices ranging from bargains over violence restraint to coordinated actions toward shared objectives, such as jointly ruling territory, protecting mutually beneficial illicit economies, and targeting common enemies. But cooperation is not always possible, for example, where nonstate and state forces control distinct territories and compete for dominance across clearly defined battle lines. Such different arrangements characterize the period between 2002 and 2006 when the Sri Lankan government and the Liberation Tigers of Tamil Eelam (LTTE) agreed to reduce violence and the faceoff between these actors toward LTTE’s destruction in 2009. This results in a variety of “wartime political orders,” from more to less to noncooperative, that exist in contexts of civil war, which determine “who rules, where, and through what understandings” (Staniland, 2012: 247).

Similarly, competing armed groups and other violent actors, including warlords (Mukhopadhyay, 2014), criminal organizations (Idler, 2019), and private militaries (Faulkner et al., 2019), not only confront each other military but also forge alliances and cooperate toward mutual objectives. For example, Christia (2012) shows that “alliance formation is tactical, motivated by a concern with victory and the maximization of wartime returns as anticipated in the political power sharing of the postconflict state” (p. 6). However, considerations other than power, particularly ideology, can equally draw competing groups to make alliances, as Gade et al. (2019) demonstrate in the case of Syria. Still, competition between armed groups formed independently of each other (Walter, 2019) and infighting between factions within and splinter groups that arise from the same movement (Bakke et al., 2012) are common features of civil wars.^10^ The nature of local orders that emerge between armed and unarmed actors during the war cannot be understood without integrating this range of intergroup dynamics into the analysis. As Balcells (2017) illustrates in the Spanish Civil War and more recent cases, such as Côte d’Ivoire, civilians might collaborate with an armed group out of a desire for revenge, to settle scores with a rival group previously in control of their territory that victimized their relatives and friends.

Finally, international actors—foreign states, diasporas, transnational insurgents, international nongovernmental and intergovernmental organizations, and private corporations—develop an array of relations with both armed and unarmed actors and impact the dynamics of wars. Over a half of nonstate armed groups since 1945 have organized transnationally, seeking neighboring state sanctuaries where they can operate out of sight of domestic rivals (Salehyan, 2009). This and other forms of support are, in turn, shaped by such considerations as international rivalries between host states and potential patrons, armed groups’ ability to pose a threat to the state, and competing armed groups’ transnational constituencies (Salehyan et al., 2011).^11^ While augmenting armed groups’ resources, such support can impose constraints on these groups’ behavior, for example, regarding tactics against the state and civilians (Moore, 2019; Petrova, 2019). It also has repercussions on armed groups’ ability to mobilize support (Bakke, 2014), their cohesion and fragmentation (Tamm, 2016), and their decisions to form alliances with each other (Bapat and Bond, 2012). The impact of international actors on the dynamics of the Syrian civil war is a vivid example.

At the same time, actors engaged in conflict transformation, above all United Nations peacekeepers, often succeed in using their resources to protect civilians, reduce violence between rivals, and contain violence geographically (Howard, 2019; Hultman et al., 2019).^12^ Internal mission composition and interactions between mission leadership at different levels and troops on the ground as well as with local populations underlie these and other outcomes (Bove et al., 2020). Other forms of third-party involvement, such as mediation, even when mediators struggle to contribute to intended national-level negotiated settlements, can reinforce peacekeeping effectiveness at the local level, including “by creating lulls during which negotiations can occur” (Beardsley et al., 2019: 1683).^13^ Civilians sometimes turn to these and other international actors to access basic necessities and services not provided by the state or armed groups (Mampilly, 2011), advance local peace initiatives (Autesserre, 2021), and protest armed actors (Kaplan, 2017), that is, to exercise agency. Yet international actors and their activities can be co-opted by armed groups to bolster their legitimacy and divert resources intended for civilian populations to their advantage, with implications for violence against civilians and humanitarian actors themselves, as in the case of Somalia.^14^

## Transformation of conflict and violence

The wartime internal organization of armed groups, their coercive and institutional interactions with civilians, and conflictual and cooperative relations with nonstate, state, and international actors extend into the post-war period as conflict and violence transform after the signing of peace agreements, military victories, and other developments that halt wartime hostilities. Most civil wars take place in countries with a history of armed conflict (Walter, 2015). However, other forms of post-war violence short of civil war are also widespread in post-war environments.^15^ Organized violence after the war can be traced to not only wartime but also pre-war and post-war dynamics. Remobilization of nonstate armed groups after the signing of peace agreements, Daly (2016) finds, has roots before the war. Those groups with strong pre-war networks that recruited participants from the area where they operated, remain geographically clustered, cohesive, and able to gather information on their former members and other armed groups. In contrast, those with weaker pre-war networks that recruited from outside of their area of operation disperse after surrendering their weapons, which prevents them from monitoring their former members and assessing their power in relation to other armed groups. After the peace agreements signed between 2003 and 2006 in Colombia, for example, in post-war areas where all armed groups were local, these groups sustained their power and demobilized over time, whereas areas with a disbalance between armed groups created by variable cohesion within these groups saw a return to violence.

Yet shared identities formed during the war and reinforced by continued relationships as part of ex-combatant communities in the post-war period can also provide the basis for ex-combatants’ return to violence. As Themnér (2011) shows in the case of the Congolese Cobra and Ninja ex-combatants, these identities are unlikely to be mobilized without the resources and leadership necessary to coordinate organized violence, no matter how marginalized ex-combatants are after war. Domestic and regional elites provide such resources and leadership as entrepreneurs of violence, commonly referred to as “spoilers” of peace (Stedman, 1997: 5). Connecting these actors to ex-combatants are intermediaries, such as former mid-ranking commanders who develop the skills and standing during the war and maintain relationships with their former subordinates that allow them to remobilize ex-combatants.

This suggests that violence in the aftermath of war is not always “a *legacy of the war*, meaning that either the actors that perpetrate or the conditions that foster the violence were created by the civil war” (Bara et al., 2021: 4, emphasis in original). The activities of armed actors continuously evolve in ways that diverge from wartime goals and strategies as “the arrival of the post-conflict period reorders the incentives and organizational structures of the combatants, thus transforming the very nature of the violence that occurs” (Boyle, 2014: 4). This reordering can fragment exiting armed groups and forge new forms of competition between armed actors, especially where negotiated settlements transfer power in ways that disrupt existing hierarchies and sideline parties with stakes in the process (Sriram and Zahar, 2009). New actors and conditions develop alongside continued violence directly related to the war (Campbell et al., 2017).^16^ For example, the 2016 peace agreement with the FARC shifted the activities of existing groups, such as the National Liberation Army (ELN) that was not part of this process, and created new groups, such as FARC dissidents that disagreed with it.

Along with such continuities and discontinuities in violence, transformations of armed groups into political, social, and economic actors, civil war legacies for affected populations, and ongoing international influence characterize the post-war period. Similarly to violence in the aftermath of war, rebel-to-party transformations have a range of sources, from pre-war experience of armed groups in electoral environments that can help these groups adapt to post-war party politics (Manning and Smith, 2016), to wartime organizational structures of armed groups, particularly governance, social service, and political wings that help repurpose these groups into political parties (Zaks, 2017), the ways in which wars end, including power-sharing arrangements in political settlements and involvement of international actors, and post-war divisions within political parties that emerge from armed groups, especially over leadership, identity, and ideology (Ishiyama and Batta, 2011).^17^ These transformations are nonlinear and often result in “hybrid politico-military organizations,” such as the Palestinian Hamas or the Lebanese Hezbollah, rather than merely *political* parties (Berti, 2013: 6).^18^

But high-ranking ex-combatants who sometimes turn into elected politicians are not the only “transitional subjects” in the post-war period (Theidon, 2007: 74). Rank-and-file ex-combatants have other experiences, from extreme poverty, lack of opportunities, and wage labor in some contexts (Utas, 2005) to participating in community organizations (Kaplan and Nussio, 2018a) and integration into state security institutions (Hartzell and Hoddie, 2020) in others.^19^ Further disaggregating the category of the armed group shows that not only high- but also mid-ranking ex-commanders continue exercising authority in formal and informal ways after the war, especially where they maintain ties to local populations in the areas where they provided services during the war (Martin et al., 2021). These ex-rebels’ authority coexists with other actors in the formation of post-war local orders. For example, extralegal groups, Cheng (2018) finds, have different motivations for governance than those of politically-driven rebel groups. They seek not to take over the state or implement political projects but to accumulate profit. Still, by providing basic governance functions needed for stable commercial environments, these groups contribute, even if unintentionally, to post-war state-building. This is evident in the case of Liberia where wartime interactions impacted the emergence of extralegal groups that then persisted into the post-war period.

Civilians participate in these arrangements affected by pre-war, wartime, and post-war dynamics. Civil wars leave long-term legacies for political identities (Balcells, 2012). Where local political networks created and maintained collective memories of the violence of the Spanish Civil War, for example, rejection of the perpetrator’s political identity endured and was mobilized in the long run (Villamil, 2021). Wood (2008) shows how wartime political mobilization, military socialization, and polarization of identities shape such networks in the first place, while Shesterinina (2021) traces the origins of post-war identities to pre-war conflict, particularly experience of intergroup violence, which polarizes and militarizes society before the war. Similarly, Huang (2016b) finds that pre-war dynamics of mobilization and organization, which shape how armed groups engage with civilian populations, pave the way for these populations’ post-war mobilization. In particular, where armed groups establish institutions of rebel governance, the populations that as a result participate in politics during the war develop an awareness of their rights and organizational capacity to mobilize for their rights thereafter, thus contributing to democratization. Mobilization after the Nepalese Civil War is exemplary. Wartime dynamics also combine with post-war developments to shape political participation after the war. Wartime killing and displacement, destruction of infrastructure and arrival of humanitarian aid, and reconceptualization of gender roles shift traditional gendered power relations to enable women’s participation in informal politics. But it is post-war regime change that springboards some women to participate in more formal capacities when new gender-sensitive norms are introduced, as in Rwanda (Berry, 2018).

Such continuities and discontinuities across the stages of conflict are also evident in the involvement of international actors in post-war societies. For example, wartime mediation is intricately related to post-war peace. Third-party mediators can help belligerents reach an agreement that otherwise would not be possible during the war, but in the long run, their involvement, especially when based on leverage, can “introduce artificial incentives for peace that do not persist, interfere with the ability for the actors to fully understand the bargaining environment, and enable the belligerents to stall in hopes of gaining an advantage during the peace process” (Beardsley, 2011: 7). More generally, third parties with vested interests in peace are those that sustain their involvement in conflict-affected countries from mediation, peacekeeping, and other wartime activities to post-war peace processes and use opportunities to learn from experience, access to local information, and relationships that they develop to shape their roles as trustworthy and effective conflict managers after the war (Chen and Beardsley, 2021). These actors’ post-war roles range from peace agreement implementation to institutionalization of peace, including through democratization (e.g. Stedman et al., 2002).

The impact of these activities, however, does not only depend on international actors’ sustained and invested involvement but also on other stakeholders’ engagement with these activities. For example, while appearing to cooperate with peacebuilding efforts, Lake (2017) demonstrates, wartime elites can use post-war institutions promoted by international actors, particularly the rule of law, to advance their wartime agendas in new ways, similarly to the co-optation of humanitarian activities by armed actors during the war. Rather than building trust, these efforts, thus, undermine the benefits they intend to deliver. From the Democratic Republic of Congo to East Timor, wartime elites engage in strategic cooperation along with conflictual interactions to solidify their individual positions and organizational advantage in the course of international institution-building efforts. Institution-building can be equally undermined by post-war state leaders (Joshi, 2013) and international efforts can face broader local resistance (Mac Ginty, 2011), especially when external actors misunderstand local contexts (Autesserre, 2014) and evade local ownership and accountability (Campbell, 2018). As Cronin-Furman and Krystalli (2021) show, these efforts can also be strategically adapted by individuals, not least victims of armed conflict, as they navigate post-war politics, for example, in the context of transitional justice in Colombia and Sri Lanka.

## Implications

From pre- to post-war periods, therefore, nonstate, state, civilian, and international actors interact, generating dynamics of conflict. In turn, the actors themselves form and transform through these dynamics. Before the war, the dynamics of mobilization and organization for war link nascent nonstate armed groups to their internal and external bases of support, other groups, and the state, shaping the nature of armed organizations that emerge during the war and their relations with civilian and other actors. Yet intragroup socialization, intergroup competition, and cooperation, including with nonstate and state forces, civilian responses to armed actors, and external influence also structure these relations. After the war, these intersecting pre-war and wartime dynamics feed into belligerents’ return to organized violence and emergence of new armed actors and violent activities, demobilized combatants’ reintegration, including through power-sharing at the top level, and transformation of networks, identities, and political engagement of war-affected populations as well as international efforts to maintain their presence in post-war settings. However, post-war-specific dynamics as well contribute to the changing landscape of actors and their activities.

This reveals crucial continuities and discontinuities between the dynamics of conflict that underlie overarching trajectories of civil wars. This article highlights some linkages between the dynamics across these periods, for example, between pre-war mobilization and organization for war and wartime internal cohesion of armed groups and between wartime institution-building by armed groups and post-war mobilization of civilians exposed to these institutions. But it also pinpoints the dynamics endogenous to wartime and post-war periods that can alter such linkages. For example, international support can undermine the internal cohesion of armed groups and political institutions introduced after the war and can enable some but not other forms of post-war mobilization. Civil wars, as a result, are complex social processes that unfold in path-dependent and endogenous ways from pre- to post-war periods.

The account of civil war as a social process advanced in this article has important implications for the study of civil war. First, it shows that civil wars develop from different pre-war dynamics of mobilization and organization, and this matters for overarching trajectories of civil wars. Whether armed groups form from a small, clandestine core of dedicated recruits, politicized networks, social movements, and/or fragmentation within the regime conditions their internal politics, interactions with civilians, ability to pose a threat to the state, and a range of other wartime and post-war outcomes that are central to the study of civil war. Scholars have recognized these path-dependent effects, as evidenced in the efforts to systematize the study of rebel group emergence, for example, through the creation of the Foundations of Rebel Group Emergence (FORGE) Dataset (Braithwaite and Cunningham, 2020). Future research should focus on how these varied origins of armed groups impact the evolution of civil wars. For example, how do initially small, clandestine groups engage in violence against civilians differently from those that develop from broad-based social movements or splits within the state military?

However, future studies should also explore how the dynamics set in motion by pre-war mobilization and organization intersect with other dynamics that are endogenous to wartime and post-war periods. This article shows that the contingency of civil wars results from the interaction of multiple actors in the course of these wars. How internal relations evolve within armed groups, civilians respond to these groups’ projects, competing nonstate and state forces engage with these groups, and international actors intervene can shift overarching trajectories of civil wars in unexpected ways. To grasp the intersection between path-dependent and endogenous dynamics over time, scholars should look at not only how actors form but also how they transform as they relate to one another. This can help better understand whether the sources of key civil war outcomes extend to pre-war, wartime, and/or post-war dynamics and when policy interventions should be made to redress these outcomes. For example, while pre-war social movement mobilization and other, including everyday, repertoires shaped Abkhaz groups that mobilized in the Georgian-Abkhaz war of 1992–1993, these groups transformed into an army in response to wartime activities of Georgian forces and external support, to achieve a military victory in the war, which otherwise would not be possible, with the contestation of this outcome characterizing post-war developments (Shesterinina, 2021). Actor-centered, relational analysis can enable scholars to access such transformations.

Methodologically, this entails paying close attention to how actors perceive and engage with one another. As this article demonstrates, not only instrumental, cost-benefit calculations but also ideational motivations rooted in social ties and identities drive actors’ interactions in civil wars. While structural analysis can help establish broad patterns of interaction, understanding actors’ internal decision-making requires a focus on the meanings the very participants attribute to their lived experiences. Such grounded knowledge can be developed through sustained engagement with people involved in conflict in different ways, from ex-combatants, to residents of war-affected areas, state officials, and members of civil society organizations and international agencies. When firsthand data collection is not ethically or logistically possible, existing primary and secondary materials, from memoirs to document archives, can give a window into participants’ perception of the events. Because these materials capture different, often competing perspectives on conflict, the ways they are generated should be part of analysis alongside efforts to leverage wide-ranging materials while recognizing their foundations in conflicting agendas. Detailed case studies informed by recognition of actors’ own understanding of their relations with one another can help get at contingent regularities rather than generalized sequences in civil wars. “Comparisons with an ethnographic sensibility” across cases can advance the accumulation of knowledge (Simmons and Rush Smith, 2021: 231).

Overall, the approach to civil war as a social process can help chart overarching trajectories of civil wars through a focus on continuities and discontinuities in the dynamics that relevant actors collectively produce as they interact with one another from pre- to post-war periods. The conceptual framework that emerges from this approach can serve as a useful analytical tool in research and policy as it identifies the actors that the analysis should focus on and the dynamics that are central to the evolution of civil wars.

## Notes

1 Civil wars have been commonly defined as wars taking place within sovereign states between nonstate and state armed forces, typically over political control of the state or part of its territory. The concept of civil war remains contested, however. On operationalization of civil war in quantitative research, see Sambanis (2004). Threshold-based definitions make a distinction between “wars” that count more than 1000 battle deaths per year and “conflicts” with at least 25 battle deaths per year (Blattman and Miguel, 2010). For a critique, see Cramer (2002) and Campbell et al. (2017), among others. For further conceptualization, see Kalyvas (2006).

2 Existing efforts to chart the dynamics of civil war focus on specific dynamics, such as the dynamics of violence (Kalyvas, 2008b), social mobilization, violence, and their legacies (Wood, 2015), and organizational dynamics (Parkinson and Zaks, 2018), or address different phases but look only at “within-war dynamics” (Cederman and Vogt, 2017: 2006), rather than a range of dynamics from pre- to post-war periods of conflict.

3 On the role of ideology in armed conflict, see Leader Maynard (2019).

4 For a review of the literature on emotions in violent mobilization, see Costalli and Ruggeri (2017).

5 On different pathways from state repression to civil war, see Davenport et al. (2006).

6 On commitment problems in civil war, see also Walter (2002).

7 On socialization, see also special issue edited by Checkel (2017).

8 In contrast, Kalyvas (2006) focuses on violent interactions in his “control-collaboration” model. On rebel governance, see also Mampilly (2011), Arjona et al. (2015), and Stewart (2021). Specifically on taxation, see Huang (2016b) and Revkin (2020), and on justice institutions, see Baczko et al. (2018).

9 For an overview of civilian strategies in civil war, see Barter (2014) and Masullo (2021). On displacement, see Steele (2017). On self-protection, see Baines and Paddon (2012) and Jose and Medie (2015). Anthropologists have long studied how ordinary people live through and navigate complex civil war environments (Lubkemann, 2008; Nordstrom, 1997; Richards, 2005).

10 On armed group fragmentation, see special issue edited by Pearlman and Cunningham (2012).

11 On transnational dynamics of civil war, see Checkel (2013). On rebel diplomacy aimed at securing external support, see Huang (2016a).

12 However, peacekeeping failures and particularly sexual violence by peacekeepers should be taken into account. For a review of the literature on peacekeeping effectiveness, see Di Salvatore and Ruggeri (2017).

13 On when and why specific rebels enter into negotiations, see Cunningham and Sawyer (2019). On the importance of linking local- and national-level dynamics, see special issue edited by Balcells and Justino (2014).

14 For a review of the literature on humanitarian aid and civil war, see Findley (2018). On the effects of international humanitarian law on civil war dynamics, see, for example, Stanton (2016) and Sutton (2021).

15 For an integrated approach to civil war recurrence and post-war violence, see Bara et al. (2021).

16 On violence against ex-combatants, for example, see, Kaplan and Nussio (2018b).

17 On rebel-to-party transformations, see also special issue edited by Ishiyama (2016).

18 On networks enabling militias-turned-parties’ mobilization for violence, see Rizkallah (2017).

19 On rank-and-file ex-combatants, see Theidon (2007). On their reintegration and recidivism, see Kaplan and Nussio (2018a, 2018b), respectively.
